# The mitochondrial pentatricopeptide repeat protein EMP12 is involved in the splicing of three *nad2* introns and seed development in maize

**DOI:** 10.1093/jxb/ery432

**Published:** 2018-12-07

**Authors:** Feng Sun, Zhihui Xiu, Ruicheng Jiang, Yiwei Liu, Xiaoyan Zhang, Yan-Zhuo Yang, Xiaojie Li, Xin Zhang, Yong Wang, Bao-Cai Tan

**Affiliations:** 1 Key Laboratory of Plant Development and Environmental Adaptation Biology, Ministry of Education, School of Life Sciences, Shandong University, Qingdao, China; 2 Agricultural Genomics Institute, Chinese Academy of Agricultural Sciences, Shenzhen, China

**Keywords:** EMP12, maize, mitochondrion, PPR, RNA splicing, seed development

## Abstract

Plant mitochondrial genes contain *cis*- and *trans*-group II introns that must be spliced before translation. The mechanism by which these introns are spliced is not well understood. Several families of proteins have been implicated in the intron splicing, of which the pentatricopeptide repeat (PPR) proteins are proposed to confer the substrate binding specificity. However, very few PPRs are characterized. Here, we report the function of a P-type PPR protein, EMP12, and its role in seed development. EMP12 is targeted to mitochondria. Loss-of-function mutation in *Emp12* severely arrests embryo and endosperm development, causing embryo lethality. The *trans*-splicing of mitochondrial *nad2* intron 2 and *cis*-splicing of *nad2* intron 4 are abolished, whereas the *cis*-splicing of *nad2* intron 1 is reduced in *emp12* mutants. As a result, complex I assembly is disrupted, and its activity is strongly reduced in the mutants. The expression of the alternative oxidase and several components of other mitochondrial complexes is increased, possibly in response to the defective complex I. These results suggest that *Emp12* is required for the *trans*-splicing of *nad2* intron 2 and *cis*-splicing of *nad2* introns 1 and 4, and is important to complex I biogenesis, and embryogenesis and endosperm development in maize.

## Teaser

The mitochondrial PPR protein EMP12 is required for the splicing of *cis*- and *trans*-introns of *nad2*, complex I biogenesis, and seed development in maize.

## Introduction

Plant mitochondrial genes have prokaryotic characteristics resulting from their origin from endosymbiosis of α-proteobacteria. Subsequently, they evolved novel features of RNA metabolism to adapt to the eukaryotic host cell environments. Mitochondria have lost most of the bacterial genes or transferred genes to the nucleus of the host cell during evolution (Gray *et al.*, 1999; Hammani and Giegé, 2014). Therefore, the mitochondrial genome retains only a small percentage of genes, encoding proteins, tRNAs, and rRNAs that are essential for the oxidative phosphorylation system (OXPHOS) and the translation machinery (Richardson *et al.*, 2013). The maize mitochondrial genome contains 58 genes, 18 of which encode subunits of complex I, III, IV, and V (*atp1* double copies) in the OXPHOS system, 4 are involved in the cytochrome *c* maturation process, 9 genes encode ribosomal proteins, and 21 genes encode tRNAs required for 14 amino acids. In addition, there are three *rRNA* genes (*rrn5*, *rrn18*, and *rrn26*), a maturase (*mat-r*) gene residing within the fourth intron of the *nad1* transcript (Clifton *et al.*, 2004), and a transporter gene (*mttB*) in the genome (Clifton *et al.*, 2004). Some genes are transcribed as long polycistronic RNA precursors. To form mature transcripts, these precursor RNAs undergo extensive post-transcriptional processing including RNA editing, which converts the cytidines to uridines (C-to-U) (Takenaka *et al.*, 2013*a*); intron splicing that removes the *cis-* and *trans*-introns and joins the exons (Brown *et al.*, 2014); RNA maturation that trims the 5' or 3' end of precursor mRNAs, and translation regulation that is facilitated by specific RNA-binding proteins (Colas des Francs-Small, *et al.*, 2014; Haïli *et al*., 2016).

Some mitochondrial genes are interrupted by introns. Based on the structure and splicing mechanism, these introns are classified as group I or group II introns, the latter of which are prevalent in plant mitochondria (Bonen, 2011). Group II introns are large ribonucleoproteins consisting of a catalytic RNA (ribozyme) and an intron-encoded maturase protein with reverse transcriptase activity (Novikova and Belfort, 2017). Structurally, group II introns have six domains (DI–DVI), in which DI, DV, and DVI are essential for splicing (Novikova and Belfort, 2017). There are 19 group II introns in the genes of *nad1*, *nad2*, *nad4*, *nad5*, and *nad7* (encoding components of complex I), and 3 introns in *cox2* (cytochrome *c* oxidase 2 of complex IV), *ccmFc* (component of cytochrome *c* maturation), and *rps3* (protein translation) in the maize mitochondrial genome (Clifton *et al.*, 2004; Brown *et al.*, 2014). Most introns are in the *cis* configuration, but some are in *trans* which require *trans*-splicing. *Trans*-introns are believed to result from the DNA rearrangement, causing a break in DIV of the intron and splitting the transcript into two, such that the two exons with the flanking half-intron are transcribed independently in the genome (Malek and Knoop, 1998; Bonen, 2011).

In contrast to bacteria, plant mitochondrial group II introns have lost the activity of self-splicing because of degeneracy and loss of the cognate maturase, leaving only an immobile maturase gene (*matR*) encoded in *nad1* intron 4 (de Longevialle *et al.*, 2010; Sultan *et al.*, 2016). To facilitate the splicing, nucleus-encoded RNA-binding cofactors are recruited, which are from different protein families. For instance, the plant organellar RNA recognition (PORR) protein, WTF9, is required for the splicing of *rpl2* and *ccmFc* introns (Colas des Francs-Small *et al.*, 2012). Similarly, the REGULATOR OF CHROMOSOME CONDENSATION-like protein, RUG3, is associated with the splicing of *nad2* intron 2 and intron 3 (Kühn *et al.*, 2011). A member of the mitochondrial transcription termination factor (mTERF) protein family, mTERF15, is involved in the splicing of the *nad2 cis*-intron 3 (Hsu *et al.*, 2014). Moreover, a chloroplast RNA splicing and ribosome maturation (CRM) protein, mCSF1 (Zmudjak *et al.*, 2013), a putative DEAD-box RNA helicase PMH2 (Köhler *et al.*, 2010), an RAD-52-like protein ODB1 (Samach *et al.*, 2011), and two nucleus-encoded maturases (Keren *et al.*, 2009, 2012) are required for the splicing of mitochondrial introns. In addition to these splicing factors, the prevalent RNA-binding proteins are from the large family of pentatricopeptide repeat (PPR) proteins (Barkan *et al.*, 2014).

PPRs belong to the α-solenoid superfamily of helical repeat proteins, with a large number in nearly all eukaryotic lineages (Fujii and Small, 2011; Barkan and Small, 2014). The structure of PPRs is defined as tandem repeats of a degenerate 35 amino acid repeat motif and a right-handed superhelix that facilitates RNA binding (Ke *et al.*, 2013; Yin *et al.*, 2013; Barkan *et al.*, 2014). PPR proteins are divided into the P- and PLS-subfamily based on the diversity of the C-terminal motifs. The P-subfamily contains the canonical P-motif, whereas the PLS-subfamily additionally harbors longer (L) or shorter (S) variant PPR motifs and additional C-terminal domains (E, E+, and DYW) (Lurin *et al.*, 2004). In land plants, >450 PPR proteins have been found (Fujii and Small, 2011) and they are mainly localized to plastids and mitochondria (Colcombet *et al.*, 2013). Some mitochondrial PPR proteins have been functionally characterized in Arabidopsis, *Physcomitrella*, rice, and maize (Barkan and Small, 2014; Colas des Francs-Small and Small, 2014). The P-subfamily PPRs usually facilitate RNA intron splicing (Brown *et al.*, 2014; Colas des Francs-Small, *et al.*, 2014; Hsu *et al.*, 2014; Hsieh *et al.*, 2015; Xiu *et al.*, 2016; Cai *et al.*, 2017; Chen *et al.*, 2017; Qi *et al.*, 2017*a*; Ren *et al.*, 2017; Dai *et al.*, 2018; Sun *et al.*, 2018), RNA stability (Colas des Francs-Small *et al.*, 2014; Lee *et al.*, 2017; Wang *et al.*, 2017; Zhang *et al.*, 2017) or RNA cleavage and translation (Colas des Francs-Small *et al.*, 2014; Haïli *et al*., 2016), whereas the PLS-subfamily is predominantly involved in RNA editing (Takenaka *et al.*, 2013*a*; Barkan and Small, 2014; Sun *et al.*, 2015; Qi *et al.*, 2017*b*; Wang *et al.*, 2017; Yang *et al.*, 2017; Li *et al.*, 2018) and occasionally in RNA splicing (Chateigner-Boutin *et al.*, 2011; Ichinose *et al.*, 2012). However, very few PPRs involved in intron splicing have been functionally characterized in Arabidopsis, as disruption of their functions often causes embryo lethality (Colas des Francs-Small and Small, 2014).

The Nad2 protein is similar to the MRP family of Na^+^/H^+^ antiporters and is a likely site for proton transfer in complex I (Hirst, 2013). Previous genetic evidence has pointed to the involvement of RNA helix proteins from distinctive families for splicing of *nad2* introns, including RUG3 (Kühn *et al.*, 2011) and mTERF15 (Hsu *et al.*, 2014) in Arabidopsis, loss of function of which results in reduced complex I activity and retarded growth. The maize kernel mutants are ideal materials to study embryo-lethal genes because of the large size and availability of homozygous endosperms and embryos. Currently, three PPR-mediated *nad2* intron splicing events in maize have been described (Xiu *et al.*, 2016; Cai *et al.*, 2017; Dai *et al.*, 2018). Maize EMP16, a P-subfamily of PPR proteins, harboring 11 PPR motifs, is involved in the *cis-*splicing of *nad2* intron 4, the lack of which precludes its normal intron splicing and complex I assembly, and, in turn, probably the defect in embryogenesis and endosperm development (Xiu *et al.*, 2016). Mutation of another P-subfamily PPR protein, EMP10, causes loss of *nad2* intron 1 splicing, which severely affects complex I activity, and the embryos in *emp10* are blocked in the proembryo stage, producing non-viable maize kernels (Cai *et al.*, 2017). Loss of DEK37 expression in the *dek37* mutants leads to reduced *nad2* intron 1 splicing, such that the embryogenesis and endosperm development are relatively alleviated, displaying a small kernel phenotype (Dai *et al.*, 2018).

In this study, we characterized a mitochondrial PPR protein designated EMP12 affecting the splicing of *nad2* introns in maize. Disruption of *Emp12* is lethal, giving rise to aborted embryogenesis and endosperm development. The splicing efficiency of *nad2 cis*-intron 1 and intron 4, and *trans*-intron 2 is reduced in *emp12* mutants, leading to the disassembly of complex I and a reduced complex I activity. Our results imply that EMP12 plays an essential role in *nad2* intron splicing, mitochondria functions, and embryo and endosperm development in maize.

## Materials and methods

### Plant materials

The maize kernel mutants, *emp12-673* (UFMu-02085) and *emp12-20* (UFMu-07644), in a W22 background were obtained from the Maize Genetics Cooperation Stock Center. The mutants were isolated from the UniformMu transposon tagging population and sequenced by high-throughput Mu-TAIL (thermal asymmetric interlaced) (Settles *et al.*, 2004; McCarty *et al.*, 2005). The *Mu* insertion was verified by genomic PCR amplification using EMP12-R: AAGCACACCATCTAATGTGTTATCACTATC and specific TIR8 primers (Tan *et al.*, 2011). Subsequently the PCR products were recovered and subjected to sequencing to confirm the *Mu* insertion position. The *Mu* active line was introgressed into the W22 inbred background. Primers EMP12-F, CACCATGCTCTTCCTCGTCCGGCG; and EMP12-R2, GGAGCAGGTTGTGGGTCTTCGTGC were used for detection of *Emp12* expression in different tissues. EMP12-F; EMP12-F2, AAGACCCACAACCTGCTCCTCCGTG; EMP12-F3, CACTGCGATCCATGCTGTTGGGATG; and EMP12-R were used for detection of *Emp12* expression in *emp12* mutants. *Ubiquitin* was used as an internal control that was amplified by primers Ubi-RTF, GCTGGAGGTCGAGAGTAGCGACAC; and Ubi-RTR, TTGACCTCAGCTCGTTGCTGTGG. Primers of *Ubiquitin* for qRT-PCR analysis were essentially according to Chen *et al.* (2017). Sequence data for maize *Emp12* was from the GenBank database under accession number GRMZM2G023071 and for alternative oxidases (AOXs) under accession numbers AY059646, AY059647, and AY059648.

### Subcellular localization of EMP12

The full-length coding sequence of *Emp12* was amplified from the maize cDNA of the W22 inbred line using primers EMP12-F and EMP12-R. The cDNA was cloned into pENTR/D-TOPO vector (ThermoFisher Scientific, http://www.thermofisher.com) and the binary pGWB5 vector. The fused *Emp12*-*GFP* was infiltrated into tobacco (*Nicotiana tabacum*) epidermal cells as described in Sun *et al.* (2015). The fluorescence signals of EMP12–green fluorescent protein (GFP) were detected at 28 h under the Olympus FluoView FV1000 confocal microscope (Olympus, http://www.olympus-global.com). The leaf slices expressing EMP12–GFP signals were dipped in 30 nM MitoTracker solution (ThermoFisher Scientific) at room temperature for 30 min before confocal microscope detection. The excitation wavelengths of GFP and MitoTracker (containing chlorophyll) were 488 nm and 559 nm, respectively.

### Light microscopy of cytological sections

The *emp12-673* kernels were harvested from self-pollinated heterozygous maize plants at 12 days after pollination (DAP) and 16 DAP. Sectioned kernels were fixed, dehydrated, and stained with Johansen’s Safranin O, and observed under a microscope as described previously (Liu *et al.*, 2013).

### Mitochondrial RNA transcript analysis

Total RNAs of embryo and endosperm of kernels at 12 DAP were extracted by using the TRIzol reagent (ThermoFisher Scientific), and subsequently digested with DNase (NEB, USA) and purified using the Ambion PureLink Plant RNA Kit (ThermoFisher Scientific). The cDNA was transcribed using random hexamer primers. Analyses of mitochondrial gene expression and intron splicing were performed in *emp12-673* and *emp12-20* mutants by reverse transcription–PCR (RT–PCR) and quantitative real-time PCR (qRT-PCR) using the primers listed previously (Xiu *et al.*, 2016; Yang *et al.*, 2017). qRT-PCR analyses were performed using SYBR Green Master Mix (Roche) using a LightCycler (Roche). The flanking exon–exon primers were used for detection of spliced RNA, and the exon–intron flanking primers were used for detection of unspliced RNA. The splicing efficiency is shown as a ratio of spliced to unspliced forms of each transcript in *emp12* mutants normalized to wild-type (WT) maize kernels (Colas des Francs-Small *et al.*, 2014).

### Mitochondrial protein and complexes analysis

Fresh embryo and endosperm of maize kernels between 12 and 14 DAP were ground in extraction buffer [0.3 M sucrose, 10 mM KH_2_PO_4_, pH 7.5, 5 mM tetrasodium pyrophosphate, 2 mM EDTA, 1% (w/v) BSA, 1% (w/v) polyvinylpyrrolidone 40, and 20 mM ascorbic acid] by using a porcelain mortar at 4 °C. The homogenate was filtered through two layers of Miracloth (Calbiochem Co., La Jolla, CA, USA) and centrifuged for 5 min at 3000 *g*. Crude mitochondria were obtained by centrifugation of the clear supernatant at 20 000 *g* for 15 min. The mitochondrial membrane proteins were measured by the Bradford assay (Bio-Rad) and a total of 8 µg of denatured proteins were subjected to SDS–PAGE for western blotting analysis (Sun *et al.*, 2015). A 100 µg aliquot of mitochondrial crude membrane proteins was solubilized in 1% *N*-dodecylmaltoside and separated by 3–12% blue native gel electrophoresis (BN-PAGE) (ThermoFisher Scientific) as described in Sun *et al.* (2015). The gel strips were stained by Coomassie Brilliant Blue R-250 (CBB) and in-gel nitroblue tetrazolium (NBT)-NADH as described in Meyer *et al.* (2009). The gel strips were incubated in 50 mM Tris–HCl, pH 6.8, 8 M urea, 1% (w/v) SDS, and 0.5% (w/v) β-mercaptoethanol for 30 min to denature the complexes and subjected to PVDF (polyvinylidene difluoride) membrane transfer and western blotting by incubating the antiserum against Nad9 (complex I/NADH dehydrogenase subunit 9) (Lamattina *et al*., 1993), maize cytochorme *c*_1_ (Cyt*c*_1_), Arabidopsis Cox2 (Agrisera), ATPase α-subunit (ATP-A), and AOX for detection of complex I, III, VI, and V, and total AOXs, respectively (Xiu *et al.*, 2016).

## Results and Discussion

### Embryo and endosperm development are arrested in *emp12*

**Fig. 1.**
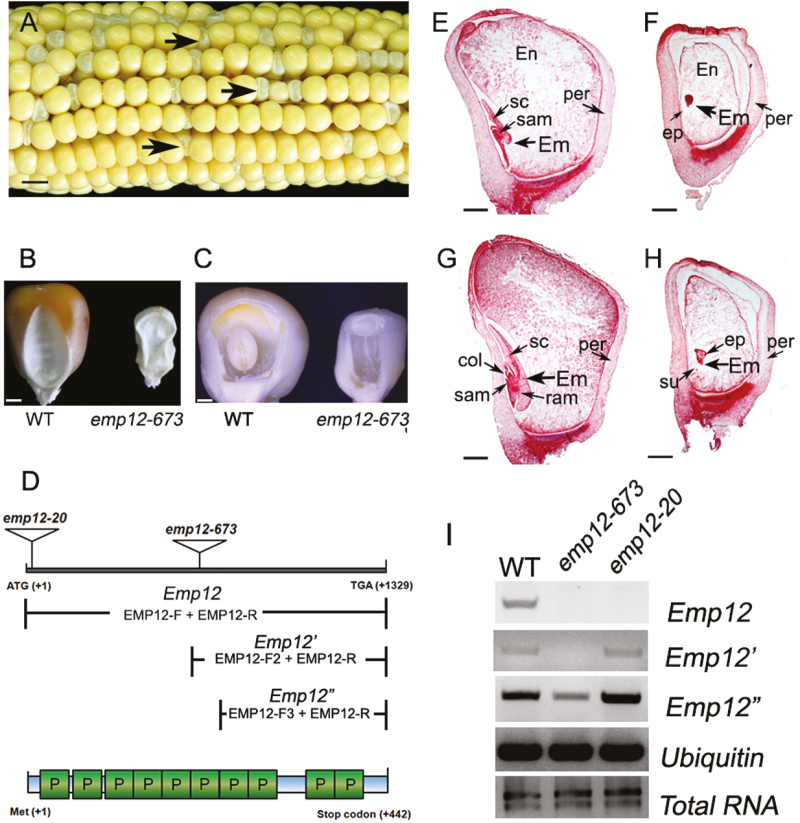
The maize *Emp12* gene is involved in embryogenesis and endosperm development. (A) A self-pollinated ear segregating for *emp12-673* mutant kernels at 15 days after pollination (DAP). Arrows show the *emp* maize kernels. Scale bar=0.5 cm. (B) The dried kernels of *emp12-673* mutants and the wild type (WT). Scale bar=2 mm. (C) The embryo and endosperm of *emp12-673* mutant and WT kernels at 12 DAP. Arrows indicate the embryo (Em). Scale bar=2 mm. (D) Schematic diagram of the *Emp12* gene and its protein structure, showing the *Mu* insertion sites of *emp12-673* and *emp12-20*. The expression of full-length and partial *Emp12* (*Emp12' and Emp12''*) downstream of the insertion sites was detected by RT–PCR analysis, with the combinations of primers EMP12-F, EMP12-F2, EMP12-F3, and EMP12-R. PPR motifs (P) of EMP12 are predicted by TPRpred (https://toolkit.tuebingen.mpg.de/#/tools/tprpred). (E–H) Light microscopy of cytological sections of WT (E, G) and *emp12-673* mutant kernels (F, H) are longitudinally sectioned early at 12 DAP (E, F) and late at 16 DAP (G, H). En, endosperm; Em, embryo; per, pericarp; sc, scutellum; su, suspensor; col, coleoptile; ep, embryo proper; sam, shoot apical meristem; ram, root apical meristem. Scale bar=1 mm. (I) RT–PCR analysis of full-length *Emp12* and truncated *Emp12'* and *Emp12''* expression indicated in (D) was performed in the *emp12-673* and *emp12-20* mutants and WT siblings at 12 DAP, with normalization by *Ubiquitin* primers.

During the screen of seed mutants from the UniformMu population (McCarty *et al.*, 2005), we identified the *emp12* mutant. The *emp12* mutant displays a severe empty pericarp phenotype (Fig. 1A–C) and cannot survive, suggesting that it is embryo-lethal. This mutant was put in the massive extraction of flanking sequences by high-throughput Mu-TAIL (Settles *et al.*, 2004). Sequence analysis indicates that the *Mu* element is inserted in the coding region 673 bp downstream of the ATG codon of a putative PPR gene (*GRMZM2G023071*) (Fig. 1D). Hence we named it *emp12-673*. The selfed progeny of heterozygous *emp12-673/+* plants displayed a 1:3 ratio [emp:(WT + heterozygotes), 140:426, χ^2^=0.02] in the WT and empty pericarp kernels, indicating that the mutation is monogenic, recessive, and nuclear. Co-segregation analysis was performed to test the linkage of *emp12-673* using *Emp12*-specifc and *Mu* TIR8 primers (Tan *et al.*, 2011). No recombination was detected from a segregating population from a self-progeny of an *emp12-673/Emp12* plant, suggesting that the *Mu* insertion is tightly linked to the *Emp12* mutation (see Supplementary Fig. S1 at *JXB* online). At 12 DAP, the *emp12* kernels were much smaller than those of the WT, displaying obscure embryo structures and vitreous endosperm (Fig. 1C). Sectioned homozygous *emp12-673* and WT kernels under microscopy indicated that the *emp12-673* mutant kernel displays a remarkable developmental retardation of the embryo and endosperm at 12 DAP (Fig. 1F). In the WT, the embryo had already formed a scutellum and shoot apical meristem, and there were clearly visible endosperm cells (Fig. 1E). At 16 DAP, the WT kernels exhibited significant growth and the pericarp clung tightly to the endosperm (Fig. 1G), while the *emp12-673* kernels grew more slowly, displaying a more crumpled empty pericarp. The *emp12-673* endosperm accumulated less starch and the embryo development stagnated at the transitional stage, remaining as an undifferentiated embryo and suspensor (Fig. 1H). Taken together, the *Emp12* mutation arrests embryo development at the transition stage and severely delays embryo and endosperm development, suggesting an essential role for *Emp12* in embryogenesis and endosperm development.

To determine whether the mutation in GRMZM2G023071 accounts for the *emp12* phenotype, another independent mutant of *Emp12* from the UniformMu population was analyzed. This *Mu* element inserted at +20 bp downstream of the ATG in *Emp12* (*emp12-20*) as indicated by the linkage and genomic PCR analysis (Fig. 1D). The selfed progeny of *emp12-20* heterozygotes separated *emp* kernels as *emp12-673*. Furthermore, an allelism test by reciprocal crosses between *emp12-673/+* and *emp12-20/+* heterozygotes produced *emp* mutant kernels, confirming that each allele could not complement each other, hence *GRMZM2G023071* is the causative gene for the *emp12* phenotype. RT–PCR amplification of the *Emp12* transcripts in these two alleles failed to detect WT *Emp12* transcripts (Fig. 1I). However, we did detect the transcripts downstream of the *Mu* insertion, suggesting the expression of the *Mu* downstream region and/or the transcript containing the *Mu* insertion. In any case, the result indicates that the WT EMP12 cannot be produced in the two *emp12* alleles. In summary, the disruption of *Emp12* results in arrested embryogenesis and endosperm development.

### EMP12 is a P-subfamily PPR protein that localizes in mitochondria

*Emp12* (*GRMZM2G023071*) is an intronless gene encoding a P-subfamily PPR protein. This PPR harbors 442 amino acids and is predicted to have 10 putative PPR motifs (Lurin *et al.*, 2004; Cheng *et al.*, 2016). EMP12 is closely related to Sb06g030430 from *Sorghum bicolor* (93%) and LOC_Os04g55090 from *Oryza sativa* (84%). However, no close homolog of EMP12 in was found *Arabidopsis thaliana* (Supplementary Fig. S2). The expression of *Emp12* is ubiquitous in a range of vegetative and reproductive tissues, showing relatively higher levels in leaves, stems, roots, silk, and developing kernels (Supplementary Fig. S3).

**Fig. 2.**
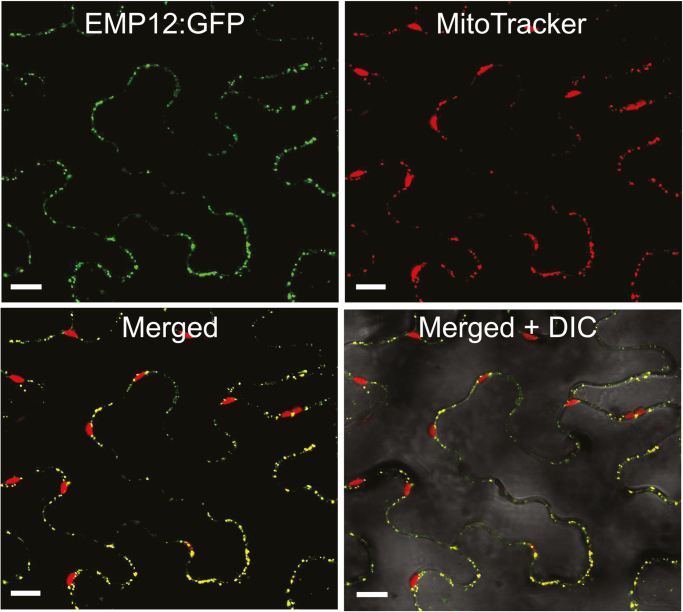
EMP12 localizes in the mitochondrion. Full-length *Emp12* was fused to green fluorescent protein (GFP) and introduced into *Nicotiana tabacum* epidermal cells, and fluorescence signals were detected under a confocal microscope. EMP12:GFP co-localizes with the specific mitochondrial marker, MitoTracker, but not with the chlorophyll fluorescence. DIC, differential interference contrast. Scale bar=10 µm.

The EMP12 protein contains an N-terminal signal peptide that is targeted to mitochondria (http://www.cbs.dtu.dk/services/TargetP/; Small *et al.*, 2004). To investigate the subcellular targeting of EMP12, the full-length *Emp12* sequence was fused to GFP and transiently expressed in *N. tabacum* epidermal cells. Confocal laser scanning microscopy indicated that the fluorescence signals of green EMP12:GFP co-localize with the specific mitochondrial marker, MitoTracker, but not with chloroplasts (Fig. 2), suggesting that EMP12 locates exclusively to the mitochondrion.

### Splicing of *nad2* introns 1, 2, and 4 is defective in *emp12*

**Fig. 3.**
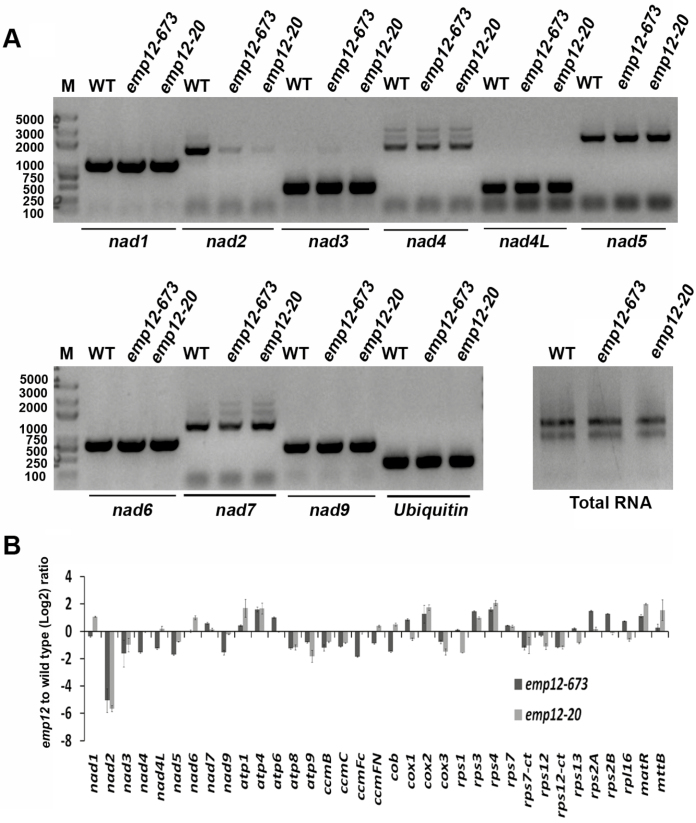
The *emp 12* mutants only affect expression of *nad2* in the mitochondria. Total RNA was extracted from fresh maize kernels at 12 DAP and reverse transcribed using hexamer primers. RT–PCR analysis was performed by using three biological replicates and was normalized to *Ubiquitin*. (A) Transcript analysis of *nad* genes in *emp12* mutant alleles. (B) Expression levels of mitochondrial transcripts were quantified by qRT-PCR analysis. The transcript abundance was plotted as *emp12*/wild-type log2 ratios using *Ubiquitin* for normalization.

EMP12 belongs to the P-subfamily PPR proteins that are involved in intron splicing, RNA stability, and RNA maturation (Barkan and Small, 2014). To gain insight into EMP12 function, the expression of mitochondrial transcripts was measured by RT–PCR and qRT-PCR analysis between the WT and *emp12* mutant kernels (Fig. 3). The results indicated that the expression of the *nad2* transcript which encodes the complex I/NADH dehydrogenase subunit2 (Nad2) was significantly reduced in the two *emp12* mutant alleles. However, no distinguishable differences were found in the expression of other mitochondrial transcripts (Fig. 3), suggesting a defective RNA processing of the *nad2* transcript in *emp12* mutants.

**Fig. 4.**
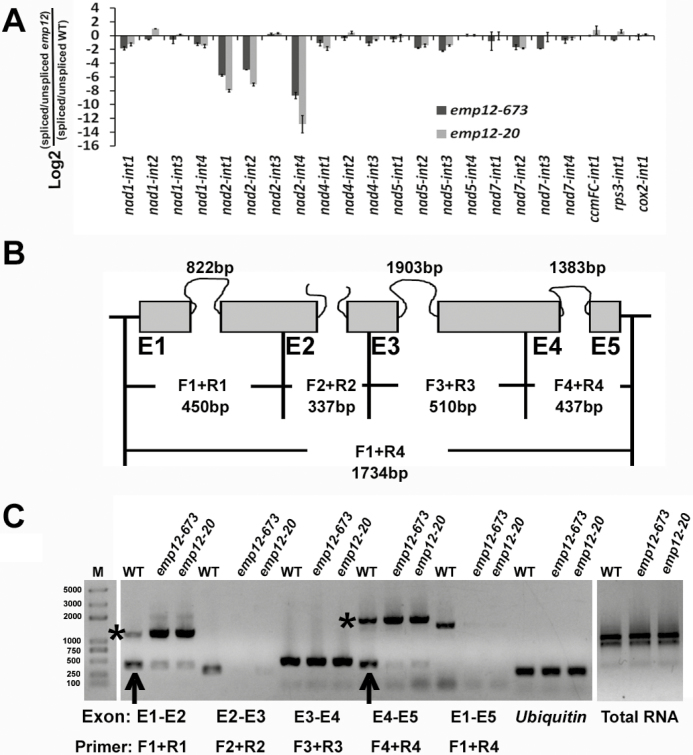
*Emp12* is required for intron 1, intron 2, and intron 4 splicing of mitochondrial *nad2.* (A) qRT-PCR analysis of all group II introns in maize mitochondrial genes. Total RNA was isolated from the *emp12-673* and *emp12-20* mutant kernels at 12 DAP. Values represent the log2 ratio of spliced to unspliced forms for each transcript in the mutants compared with WT maize kernels. Each value is the mean of at least three biological replicates. (B) Structure of the maize *nad2* gene. Exons are shown as filled gray boxes. The closed and open lines stand for *cis*- and *trans*-introns. Primers (F1+R1, F2+R2, F3+R3, F4+R4, and F1+R4) indicate the PCR products by using flanking exon–exon primers as described previously (Xiu *et al.*, 2016). E1–E5, exon1–exon5. (C) RT–PCR analysis of the intron splicing of *nad2* introns using exon–exon primers as indicated in (B). Arrows and asterisks indicate the spliced and unspliced PCR products, respectively.

The *nad2* transcript in maize contains four introns; intron 2 is *trans*-spliced while the rest are *cis*-spliced (Fig. 4B). Since *nad2* is greatly reduced in *emp12* mutants, possible splicing defects in *nad2* introns were monitored by using both exon–exon and exon–intron flanking primers (Xiu *et al.*, 2016; Yang *et al.*, 2017). The results show that in *emp12-673* and *emp12-20* alleles, the *trans*-splicing of intron 2 and *cis*-splicing of intron 4 of the *nad2* transcript were lost, whereas the *cis*-splicing of intron 1 of *nad2* was reduced (Fig. 4A), pointing to the requirement for *Emp12* in *nad2* intron splicing. Moreover, no differences were found in the introns of the other five mitochondrial transcripts (Fig. 4A). Prediction of the binding sites by the recognition code at positions 6 and 1' indicated that these three introns might share similar binding sequences that are not present in other introns (Barkan *et al.*, 2012; Takenaka *et al.*, 2013*b*) (Supplementary Fig. S4). Therefore, the reduced expression of *nad2* in *emp12* mutants is associated with the splicing defects of three *nad2* introns.

### Complex I biogenesis is reduced in *emp12*

**Fig. 5.**
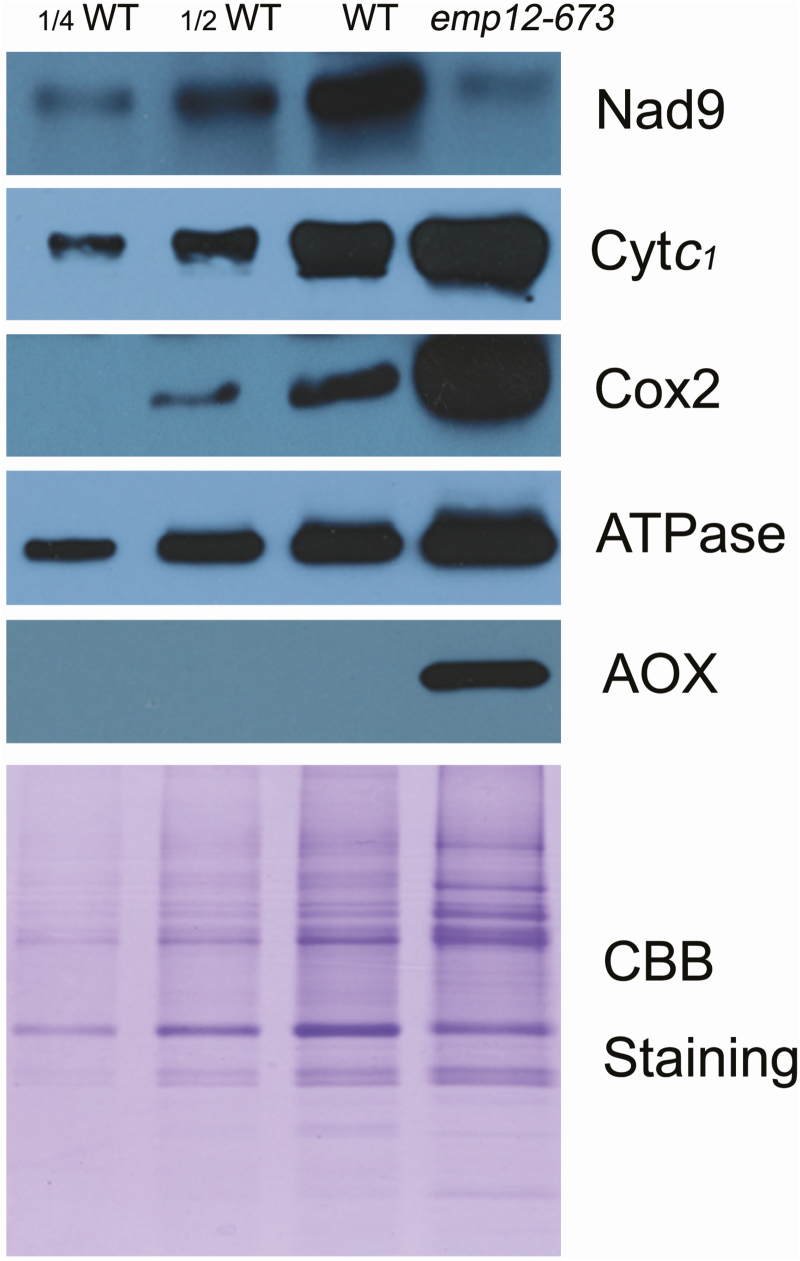
Protein abundance of the mitochondrial respiration chain is affected in the *emp12* mutants. Freshly prepared mitochondrial membrane proteins in *emp12* and WT maize kernels were subjected to SDS–PAGE, and the proteins were then transferred to a polyvinylidene difluoride (PVDF) membrane and probed with antibodies against Nad9, Cyt*c*_1_ (cytochrome *c*_1_), Cox2, Cyt*c* (cytochrome *c*), ATPase, and AOX (alternative oxidase). Coomassie Brilliant Blue- (CBB) stained gels are shown to demonstrate that equal amounts of proteins were loaded.

To gain insight into whether the splicing defect of *nad2* introns affects the function of the respiration chain in the *emp12* mutants, representative mitochondrion-encoded protein components of each of the mitochondrial complexes in the maize kernels were first monitored by western blot analysis. The results showed that the protein abundance of Nad9 (complex I/NADH dehydrogenase subunit 9) (Lamattina *et al.*, 1993), which is a peripheral membrane subunit of complex I, was severely reduced in *emp12-673*. This scenario is also seen in other *nad2* intron splicing mutants such as *emp16* (Xiu *et al.*, 2016), suggesting that the lack of *nad2* splicing results in the lost stability of peripheral proteins of complex I. In contrast, a core membrane subunit from complex III, Cyt*c*_1_, was strongly increased. In addition, Cox2 (cytochrome oxidase subunit2) from complex IV, and mitochondrial ATP synthase α-subunit from complex V, were also increased in *emp12-673* mutants (Fig. 5). A possible explanation is that the mitochondrial respiratory chain contains 92 subunits, comprising both mitochondrion- and nucleus-encoded components (Jacoby *et al.*, 2012; Subrahmanian *et al.*, 2016). In *emp12* mutants, complex I is affected, so expression of proteins from other complexes or branched electron transport chains would be enhanced to adapt to the altered electron transfer state and NADH accumulation.

**Fig. 6.**
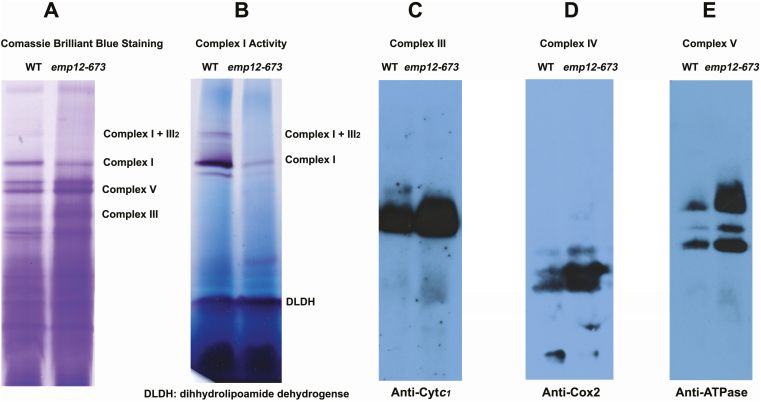
The *Emp12* mutation impairs mitochondrial biogenesis in *emp12* mutants. The crude mitochondrial membrane was freshly extracted from the *emp12* mutant kernels. The complexes were solubilized by 1% dodecyl maltoside and subjected to blue native gel electrophoresis (BN-PAGE) as described in Sun *et al.* (2015). (A) The mitochondrial membrane complexes were separated by the BN gels and stained with Coomassie Brilliant Blue. (B) Detection of NADH dehydrogenase activity of complex I. Dihydrolipoamide dehydrogenase (DLDH) was used as a loading control. (C–E) Accumulation of respiratory chain complex III, IV, and V in *emp12* mutant kernels. The BN gels were denatured, transferred onto the PVDF membranes, and probed with antibodies against Cyt*c*_1_ for complex III (C), Cox2 for complex IV (D), and ATPase (α-subunit) for complex V (E).

The assembly and amount of respiratory complexes of *emp12* were further determined by BN-PAGE using dissolved crude mitochondrial membrane proteins from embryo and endosperm. Complex I in *emp12* was strongly reduced, leaving little assembled complex I as indicated by the CBB and in-gel NBT-NADH activity staining. In addition, the supercomplex (I+III_2_) in *emp12-673* was strongly reduced, suggesting that the assembly of complex I in *emp12* mitochondria is severely impeded (Fig. 6A, B). In contrast, as shown by CBB staining and western blotting, complex III, IV, and V accumulated to levels greater than found in the WT (Fig. 6C–E). A similar shift in the relative amounts of these complexes was also noted with other complex I mutants such as *nmat1, 2, 4* mutants in Arabidopsis (Keren *et al.*, 2009, 2012; Cohen *et al.*, 2014) and *emp16* and *emp8* mutants in maize (Xiu *et al.*, 2016; Sun *et al.*, 2018).

### Alternative oxidase is activated in *emp12*

AOX drains the electrons from the ubiquinone pool, by-passing the cytochrome *c* pathway for ATP synthesis (Moore and Siedow, 1991; Kühn *et al.*, 2015). Both RT–PCR and qRT-PCR analyses indicated that among the three *AOX* genes, the *AOX2* transcript in *emp12* mutant alleles was strongly increased in comparison with the WT, whereas *AOX1* and *AOX3* showed an indistinguishable expression (Supplementary Fig. S5). Western blotting confirmed that the maize AOX proteins in the *emp12* mutant were strongly increased (Fig. 6), which is consistent with the scenarios occurring in other complex I mutants (Li *et al.*, 2014; Xiu *et al.*, 2016; Chen *et al.*, 2017; Cai *et al.*, 2017; Qi *et al.*, 2017*a*, b, Ren *et al.*, 2017; Zhang *et al.*, 2017; Dai *et al.*, 2018; Sun *et al.*, 2018). These results indicate that the alternative pathway is activated to reduce levels of reactive oxygen species (ROS) when electron flow is improperly maintained through the cytochrome *c* pathway in *emp12* mutants (Wagner and Moore, 1997).

### Splicing of one intron of *nad2* involves the co-ordination of more than one splicing factor

EMP12 was found to act on *nad2* intron 4 splicing, whereas EMP16 also specifically participates in the splicing of this intron, implying that more than one splicing factor is involved in the splicing of a specific intron (Supplementary Fig. S4B). EMP12 is also involved in the *cis*-splicing of *nad2* intron 1, which has been described to require other PPR proteins, such as EMP10 (Cai *et al.*, 2017), DEK37 (Dai *et al.*, 2018), and EMP8 (Sun *et al.*, 2018) in maize. In addition to maize, it has also been found that in Arabidopsis, splicing factors from various families, namely the PPR protein MTSF1 (Haïli *et al.*, 2013), together with the CRM protein mCSF1 (Zmudjak *et al.*, 2013), the DEAD-box protein PMH2 (Köhler *et al.*, 2010), a maturase nMAT1 (Keren *et al.*, 2012), and a RAD-52-like protein ODB1 (Samach *et al.*, 2011), are involved in the *nad2* intron 1 splicing. Other *nad2* introns, such as *cis*-intron 3, require the Arabidopsis PPR protein ABO5 (Liu *et al.*, 2010), mCSF1 (Zmudjak *et al.*, 2013), the TERF family protein mTERF15 (Hsu *et al.*, 2014), and the RUG protein RUG3 (Kühn *et al.*, 2011) for the splicing. These results suggest that splicing of a specific intron involves the co-ordination of specialized and general RNA-binding proteins. In addition, these splicing factors, particularly PPRs, showed distinct disparity (or at least lack of distinct sequence conservation) on one intron in monocots (i.e. maize) and dicots (i.e. Arabidopsis). It may reflect the evolutionary divergence and the complexity of splicing. The requirement for a splicing factor in organelles is probably a co-evolutionary result between the mutation in the intron and the corresponding recruitment of a nuclear-encoded protein such as the PPR proteins. In addition, it is also probable that the splicing of a specific intron requires multiple PPR proteins. If the mutation in the intron occurs after the divergence of monocots and eudicots, the splicing PPRs could be different in monocots and eudicots although they function on the same intron. If the mutation is prior to the divergence, the PPR proteins could be similar.

PPRs probably recognize the intron-binding sequences independently or, alternatively, they most probably form a highly dynamic complex similar to the nuclear spliceosome. Yeast two-hybrid analyses between EMP12 and EMP16 revealed no direct interaction (Supplementary Fig. S6). However, PPRs have been described to form a complex with other unknown proteins. PNM1, a dual-targeted PPR protein, has been implicated in a 120 kDa complex (Hammani *et al.*, 2011; Senkler *et al.*, 2017). GRP23 (Ding *et al.*, 2006) was recently identified in a 160 kDa complex (Senkler *et al.*, 2017) and is in a complex including PMH2 and nMAT2 in mitochondria (Zmudjak *et al.*, 2017). Two PPR proteins, DYW2 and NUWA (He *et al.*, 2017), constitute the main components of the editosome in mitochondria. They interact with the mitochondrial PPR protein SLO2 and chloroplast PPR protein CLB19 for RNA editing. NUWA is thought to act as a general bridge for the editing of SLO2 and CLB19 at specific sites (Andres-Colas *et al.*, 2017; Guillaumot *et al.*, 2017). It is most likely that EMP12 constitutes a complex similar to the highly dynamic nuclear spliceosome for each intron, either transiently or stably, as it specifically acts on three introns of the *nad2* transcript, to maintain the configuration in a ribozyme active state.

## Supplementary data

Supplementary data are available at *JXB* online.

Fig. S1. The *Mu* insertion in *Emp12* linked with the empty pericarp phenotype in the *emp12-673* allele.

Fig. S2. The amino acid alignment of EMP12 homologs.

Fig. S3. qRT-PCR analysis of *Emp12* expression in different tissues and kernels at different developing stages.

Fig. S4. Predicted binding sites of EMP12 and EMP16 in *nad2* introns.

Fig. S5. *AOX2* expression is increased in *emp12* mutants.

Fig. S6. EMP12 did not interact with EMP16 as demonstrated by yeast two-hybrid assay.

Supplementary Figures S1-S6
