# Supplementary material for: The mitochondrial pentatricopeptide repeat protein EMP12 is involved in the splicing of three nad2 introns and seed development in maize
Source: J Exp Bot. 2018 Dec 7;70(3):963–72. doi: 10.1093/jxb/ery432 (PMC6363090; doi:10.1093/jxb/ery432)
Supplement: Supplementary Figures S1-S6 [file ery432_suppl_supplementary_figures_s1-s6.pdf]

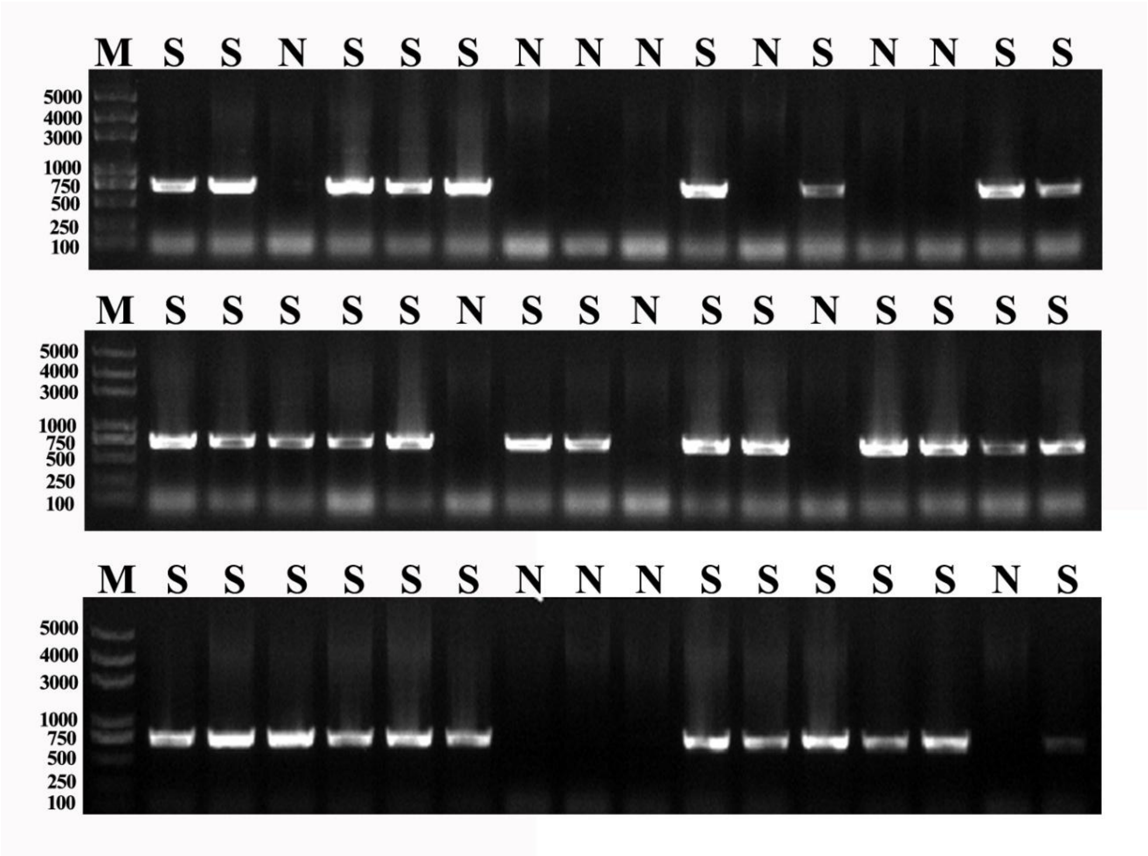

Fig.S1. The *Mu* insertion in *Emp12* links with the empty pericarp phenotype in the *emp12-673* allele.

Individual seedlings of *Emp12* heterozygotes and WT were differentiated by using *Emp12* specific primers of EMP12-R and Mu-TIR primer TIR8. PCR products at 653 bp are indicative of a *Mu* insertion at 673 bp (*emp12-673*) downstream of the ATG of *Emp12*. The *emp12* phenotype was observed in the cob after selfing. “N”: non-segregating WT plants; “S”: segregating heterozygous *Emp12/emp12* plants.

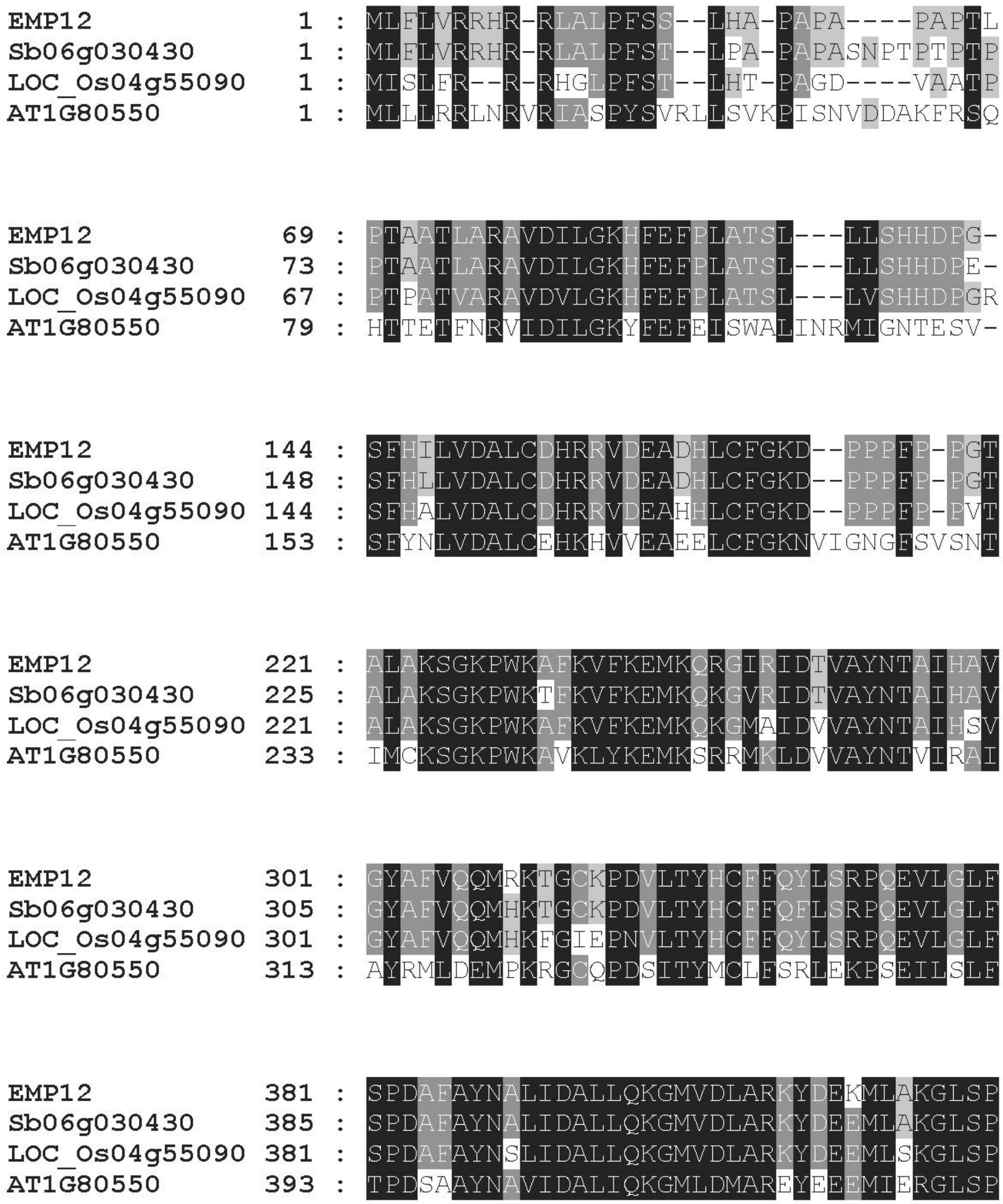

Fig.S2. The amino acid alignment of EMP12 homologs.

The amino acid sequences of EMP12 homologs were retracted from the phytozyme database (https://phytozome.jgi.doe.gov/pz/portal.html). The maize EMP12 was compared with the homologous sequences of the monocotyledonous sorghum (Sb06g030430), rice (LOC_Os04g55090) and the dicotyledonous Arabidopsis (AT1G80550).

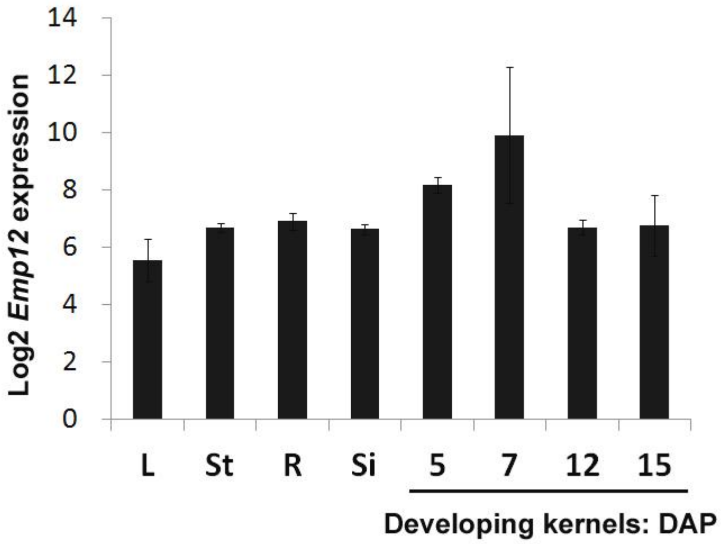

Fig.S3. Quantitative RT-PCR analysis of *Emp12* expression in different tissues and kernels at different developing stages.

The tissues of maize, including leaf (L), stem (St), root (R), silk (Si) were collected from a maize plant pending for pollination. Total RNA was extracted from at least three replicates and transcribed for gene expression analysis. *Ubiquitin* was used as an internal control. DAP, day after pollination.

A

PPR motif	P	P	P	P	P	P	P	P	P	P	Position
	A	A	R	H	N	S	N	N	V	N	6
	L	F	D	G	D	D	N	D	D	K	1’
nad2 intron 1	*g*	*c*	*a*	*g*	*c*	*u*	*c*	*u*	*u*	*u*	
nad2 intron 2	*g*	*u*	*a*	*u*	*c*	*g*	*c*	*u*	*u*	*u*	
nad2 intron 4	*g*	*c*	*a*	*g*	*u*	*g*	*c*	*u*	*u*	*u*	

B

PPR motif	P	P	P	P	P	P	P	P	Position
EMP16	N	N	N	T	S	S	G	N	6
	T	D	T	N	N	D	H	D	1’
nad2 intron 4	*c*	*u*	*c*	*g*	*a*	*u*	*g*	*u*	

Fig. S4. Predicted binding sites of EMP12 and EMP16 in *nad2* introns.

The amino acids at positions 6 and 1’ in PPR motifs of EMP12 **(A)** and EMP16 **(B)** are predicted according to Cheng *et al.,* 2016. The binding sequence of *nad2* intron 1, intron 2, and intron 4 is determined by the frequencies of co-occurrence of amino acids at positions 6, 1’ and nucleotides of introns (Barkan *et al.,* 2012; Takenaka et al., 2013b). The stronger binding of nucleotide combinations is shown by yellow color, the moderate binding is shown by green color, whereas the weak binding is shown in gray green color. “P” stands for classical PPR motifs.

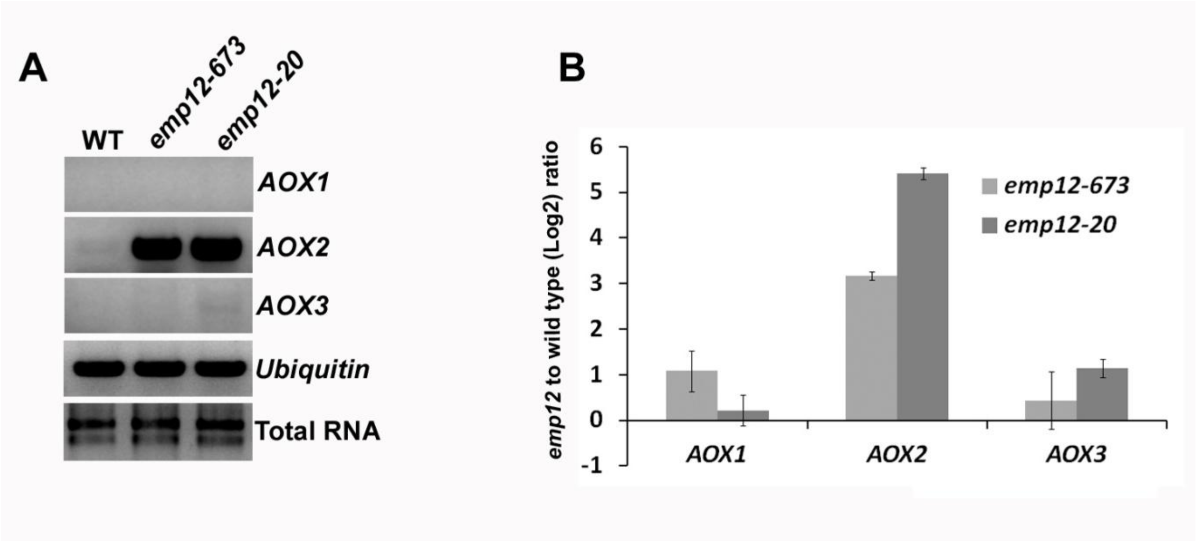

Fig. S5. *AOX2* expression is increased in *emp12* mutants.

(**A**) RT-PCR analysis of the *Alternative Oxidase* (*AOX*) gene expression of *emp12* mutant kernels at 12 DAP.

(**B**) Quantitative qRT-PCR analysis of *AOX* expression of in *emp12* mutant kernels at 12 DAP.

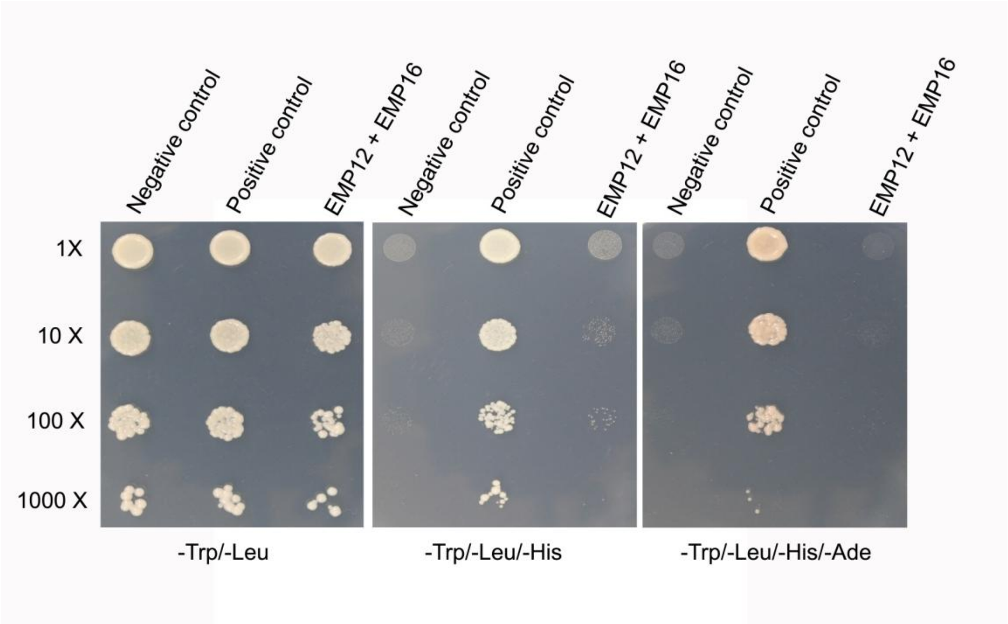

Fig.S6. EMP12 did not interact with EMP16 as demonstrated by yeast two-hybrid assay.

Mature EMP12 and EMP16 without signal peptide were co-transformed and sprayed on dropout medium of DDO (SD/-Leu/-Trp), TDO (SD/-Leu/-Trp/-His) and QDO （SD/-Leu/-Trp/-His/-Ade）. Blank vectors of BD (pGBKT7) and AD (pGADT7) and positive controls were co-transformed according to the manual of Clontech.
